# Importance of phenols structure on their activity as antinitrosating agents: A kinetic study

**DOI:** 10.4103/0975-7406.76491

**Published:** 2011

**Authors:** Márcia Pessêgo, Ana M Rosa da Costa, José A. Moreira

**Affiliations:** 1 Department of Physical Chemistry, Faculty of Chemistry, University of Santiago, 15782, Santiago de Compostela, Spain; 2 CIQA and DQF, Faculty of Science and Technology, University of Algarve, Campus of Gambelas, 8005-139 Faro, Portugal,

**Keywords:** Antinitrosating action, DNA bases, Kinetics, *N*-methyl-*N*-nitrosobenzenesulfonamides, Phenols, Transnitrosation

## Abstract

**Objective:**

Nitrosative deamination of DNA bases induced by reaction with reactive nitrogen species (RNS) has been pointed out as a probable cause of mutagenesis. (Poly)phenols, present in many food items from the Mediterranean diet, are believed to possess antinitrosating properties due to their RNS scavenging ability, which seems to be related to their structure. It has been suggested that phenolic compounds will react with the above-mentioned species more rapidly than most amino compounds, thus preventing direct nitrosation of the DNA bases and their transnitrosation from endogenous *N*-nitroso compounds, or most likely from the transient *N*-nitrosocompounds formed *in vivo*.

**Materials and Methods:**

In order to prove that assumption, a kinetic study of the nitroso group transfer from a *N*-methyl-*N*-nitrosobenzenesulfonamide (*N*-methyl-*N*-nitroso-4-methylbenzenesulfonamide, MeNMBS) to the DNA bases bearing an amine group and to a series of phenols was carried out. In the transnitrosation of phenols, the formation of nitrosophenol was monitored by Ultraviolet (UV) / Visible spectroscopy, and in the reactions of the DNA bases, the consumption of MeNMBS was followed by high performance liquid chromatography (HPLC).

**Results:**

The results obtained point to the transnitrosation of DNA bases being negligible, as well as that of phenols bearing electron-withdrawing groups. Phenols with methoxy substituents in positions 2, 4, and / or 6, although they seemed to react, did not afford the expected product. Phenols with electron-releasing substituents, unless these blocked the oxygen atom, reacted with our model compound at an appreciable rate. *O*-nitrosation of the phenolate ion followed by rearrangement of the C-nitrosophenol seemed to be involved.

**Conclusion:**

This study provided evidence that the above compounds might actually act as antinitrosating agents *in vivo*.

Exposure of humans to excess nitrosating species from the diet, cosmetics, pharmaceuticals, the environment or from overproduction of endogenous nitric oxide (NO) is commonly linked to mutagenic and carcinogenic events.[1,2] The reactive nitrogen species (RNS), which comprise of nitric oxide, dinitrogen trioxide, nitrite, nitroxyl and nitrosonium ions, and nitrous acid, are interconnected through a series of reactions that can be admitted to start either with the generation of NO from arginine by nitric oxide synthase or with the transformation of nitrite ions into nitrous acid in the acidic environment of the stomach.[1,3] It was recently proven that dinitrogen trioxide has a biological relevance, as it originates not only from the reaction of nitric oxide with oxygen, but can also be formed by the reaction of nitric oxide with a superoxide.[4]

Nitrosative deamination of the DNA bases, involving either a direct reaction with RNS or a nitroso group transfer from NO donors such as *N*-nitrosamides and *N*-nitrosamines,[5–8] can lead to mutagenesis through misincorporation by DNA polymerase, misrepair, or no repair of the resulting deamination products, which may result in the formation of abasic sites. The diazonium ions thus formed in the bases with aminic nitrogen may undergo nucleophilic substitution by water, leading to the transformation of cytosine, guanine, and adenine in uracil, xanthine, and hypoxanthine, respectively.[5–7,9,10] Published results point to guanine being more reactive than adenine toward sodium nitrite in acidic medium and to cytosine not reacting at all.[11] Moreover, *N*-nitrosamines, upon metabolic activation by cytochrome P450, form DNA oxidizing and alkylating agents.[12]

Phenolic compounds are known to act as natural antioxidants and antinitrosating agents. A regular intake of (poly)phenolic compounds widely found in fruits, vegetables, tea, and red wine is believed to decrease the incidence of certain forms of cancer, and for that reason they are commonly regarded as chemopreventive agents.[1,13] The antioxidant properties of phenols are determined by their radical scavenging ability and consequent inhibitory action on lipid peroxidation under oxidative stress conditions, which correlate with their substitution pattern.[14]

The antinitrosating activity of phenols is thought to be due to their action as RNS scavengers, thus preventing both direct nitrosation of DNA bases[11] and endogenous formation of carcinogenic *N*-nitroso compounds,[15,16] by reacting with the above-mentioned species more rapidly than most amino compounds. However, along with this long-known inhibitory effect on the formation of N-nitrosamines, phenols and some polyphenols have been reported to act as catalysts rather than inhibitors of such reactions.[17] A mechanism is proposed, in which the quinone monoxime tautomer of a nitrosophenol reacts with nitrous acid to produce an intermediate nitrosating agent, which undergoes an attack by the amine to produce *N*-nitrosamine and regenerate the catalyst,[18] although it has been stressed that catalytic activity is only observed when the nitrosating agent concentration significantly exceeds the concentration of phenol, which is rare *in vivo* or in environmentally significant situations.[19] It has also been observed that the introduction of a nitroso group in phenols brings about a loss in their antioxidant activity.[14]

The purpose of this study is to acquire a deeper understanding of the mechanism of DNA base transnitrosation by NO donors, and on the possible action of phenols in avoiding that reaction, by comparing the relative rates of the nitroso group transfer to both classes of nucleophiles, using *N*-methyl-*N*-nitroso-4-metilbenzenesulfonamide (MeNMBS) as model compound. This and other *N*-methyl-*N*-nitrosobenzenesulfonamides have been used before as nitrosating agents in the study of the transnitrosation of amines[20] and phenols.[21] In addition, we expect to collect more evidence on the mechanism of nitrosophenol formation, as there has been some discussion on whether the reaction occurs directly at the carbon atom[19,22,23] or the previous formation of an *O*-nitroso intermediate is involved.[21,24] The nitrosation of OH-blocked ‘phenols,’ such as anisole, is possible when the mechanism involves the nitrosonium ion polar reaction,[25] but it will not be observed when the reaction follows the oxidative pathway via the phenoxy radicals, because the formation of those are hindered, as observed by Daiber *et al*.[26]

We must also remark that the team of Luis García-Rio was developed a rather elegant methodology to distinguish reactivity in ambident nucleophiles. In their case they have been able to determine that for the nitrosation of enols, the reaction follows two parallel pathways, one involving C-nitrosation and the other O-nitrosation.[27]

## Materials and Methods

### Reagents and equipment

Reagent grade guanine, cytosine, adenine, uracil, hypoxanthine, guaicol (2-methoxyphenol), 3-methoxyphenol, 2-bromophenol, 3-bromophenol, 2,3-dimethoxyphenol, 2-chlorophenol, 2-fluorophenol and 2,6-di-*tert*-butylphenol, sulfanilamide and *N*-1-naphtylethylenediamine from Aldrich, xanthine from Sigma, 4-methoxyphenol, 4-chlorophenol and *N*-methyl-*N*-nitroso-4-metilbenzenesulfonamide (MeNMBS) from Merck, carvacrol (2-methyl-5-*iso*propylphenol), 4-bromophenol, 3,5-dimethoxyphenol and syringol (2,6-dimethoxyphenol) from Fluka, 3,5-di-*tert*-butylphenol from Enamine, *N*-methyl-4-metilbenzenesulfonamide (MeMBS) from Ega-Chemie were used; thymol (5-methyl-2-*iso*propylphenol) and anisole were from Riedel-de-Haën; the latter was previously distilled and the second fraction (41–42°C)[24] was used. HPLC grade acetonitrile and methanol were from Lab-Scan, and 1,4-dioxane from Riedel-de-Haën and bidistilled water from a quartz bidistiller. An UV-Vis spectrophotometer (Varian Cary 50 Bio), an HPLC (Agilent 1100) with RP-18 LiChrospher column (250 × 4 mm, 5 *µ*m), and diode array UV-Vis detector, a thermostated bath (Julabo F12), with ± 0.1°C precision and a pH meter (Thermo Orion 4 Star pH-ISE Benchtop) were used. In the kinetic studies, the experimental points were adjusted to equations using GraFit^®^ 5.0.5. (Erithacus Software Limited).

### Solutions

The following solutions were prepared: phosphate buffer 50 mM, pH 3 and 4, and 0.1 M, pH 9.5, 10, 10.25, 10.75, 11, and 11.5; guanine 1.75 × 10 ^-4^, 2.6 × 10 ^-4^, 3 × 10 ^-4^, and 3.5 × 10 ^-4^ M, in pH 3 and pH 4 phosphate buffer; cytosine 1.75 × 10 ^-4^, 2.5 × 10 ^-4^, 3.5 × 10 ^-4^, 1 × 10 ^-3^, 5 × 10 ^-3^, and 1 × 10 ^-2^ M, in pH 3 phosphate buffer; adenine 2 × 10 ^-4^, 1 × 10 ^-3^, 3 × 10 ^-3^, 5 × 10 ^-3^, 7 × 10 ^-3^, and 1 × 10 ^-2^ M, in pH 3 phosphate buffer; 0.1 and 0.3 M of each phenol in dioxane; 0.01 M of MeNMBS in dioxane; 0.01 M of 2,2,6,6-tetramethyl-1-piperidinyloxy (TEMPO) in water.

### Kinetic study of the acidic transnitrosation of DNA bases

In the study of the DNA base concentration effect on the reaction rate, the appropriate volumes of pH 3 solutions were placed in a vial in order to get the following final concentrations: guanine 1.74 × 10 ^-4^ to 3.48 × 10 ^-4^ M; cytosine 1.74 × 10 ^-4^ to 9.93 × 10 ^-3^ M; and adenine 1.99 × 10 ^-4^ to 9.93 × 10 ^-3^ M. After stabilization at 35°C for 10 minutes, MeNMBS solution was added, to a final concentration of 6.67 × 10 ^-5^ M, in a total volume of 3 mL. To determine the pH effect on the reaction between guanine and MeNMBS, the previous study was repeated at pH 4. The decay of MeNMBS concentration with time was obtained from the peak areas at 247 nm in the HPLC chromatograms of the reaction mixture. In the analysis of the reaction mixtures of guanine and cytosine, a step gradient from a sodium phosphate buffer (50 mM, pH 3), to a mixture of 40% buffer and acetonitrile was used. Retention times were: guanine 7.0 minutes, xanthine 11.2 minutes, cytosine 3.1 minutes, uracil 5.0 minutes, MeMBS 19.9 minutes, and MeNMBS 22.9 minutes. For the reactions of adenine, elution was carried by a step gradient from a mixture of 87.5% phosphate buffer (50 mM, pH 4 and 2 mM in triethylamine) with methanol to 30% buffer. Retention times were: hypoxanthine 3.7 minutes, adenine 9.3 minutes, MeMBS 15.6 minutes, and MeNMBS 17.9 minutes.

### Kinetic study of the basic transnitrosation of phenols

In the study of the phenol concentration effect on the reaction rate, appropriate volumes of solutions to get final concentrations ranging from 1 × 10 ^-3^ to 6 × 10 ^-3^ M, were pipetted to a spectrophotometer quartz cell containing pH 10.75 buffer solution. The cells were placed in the spectrophotometer and allowed to stabilize for 15 minutes at 25°C and MeNMBS solution was added to the final concentration of 1 × 10 ^-4^ M, in a total volume of 3 mL. To determine the pH effect, the study was carried in buffer solutions from pH 9.5 to 11.5. The formation of nitrosophenol was monitored by measuring the solution absorbance at the appropriate wavelength [Table 1]. The quantification of the by-product phenol *p*-toluenesulfonate was made by HPLC analysis of the reaction mixture in a 60% acetonitrile / 40% water mixture and the product distribution was established by conjugation of this data, with quantification of the initial MeNMBS, using the Shin’s method.[29] Retention times were: NO-thymol 2.7 minutes, thymol 6.7 minutes, NO-carvacrol 3.7 minutes, carvacrol 6.3 minutes, 3-methoxyphenol 2.8 minutes, 3-methoxyphenol tosylate 9.3 minutes, 3,5-dimethoxyphenol 2.9 minutes, 3,5-dimethoxyphenol tosylate 10.5 minutes, and MeMBS 3.1 minutes.

**Table 1:** 

PhOH	NO-Ph λ_max_ /nm	p***K***_a_ lit.	p***K***_a_ exp.	*k*/**M-1.s-1**
1a	416	8.45 [33a]	-	*
1b	410	8.56 [33a]	-	*
1c	404	8.70 [33a]	-	*
1d	403	9.03 [33a]	-	*
1e	410	9.17 [33a]	-	*
1f	339	9.34 [33a]	9.01 ± 0.02	(1.9 ± 0.3) × 10-3
1g	426	9.41 [33a]	-	*
1h	348	9.65 [33a]	10.29 ± 0.05	(2.8 ± 0.5) × 10-2
1i	464	9.97 [33b]	-	†
1j	392	9.98 [33a]	-	†
1k	437	10.10 [33a]	-	†
1l	428	‌‌	-	†
1m	‡	10.30 [33c]	-	
1n	390	10.38 [33d]	10.58 ± 0.14	(3.0 ± 0.7) × 10-2
1o	390	10.60 [33a]	10.71 ± 0.10	(3.4 ± 0.6) × 10-2
1p	376	11.70 [33a]	-	†

*The reaction proceeded very slowly and the data did not fit properly. A first order integrated equation; ^†^Product formation was not observed; ‡Failed to react with acidic nitrite; ^§^Impossible to solubilize in the reaction medium; ‌‌Not found in the literature.

### Synthesis of nitrosophenols and phenyl *p* -toluenesufonates

In order to determine the appropriate wavelength to follow the transnitrosation reactions, authentic samples of nitrosophenols were synthesized by acidifying a mixture of phenolate and sodium nitrite with sulfuric acid. [30a] In the reaction of 3,5-di-*tert*-butylphenol no isolable product was formed. The hindered 2,6-dimethoxyphenol was nitrosated by dissolution in an ethanolic hydrochloric acid solution, to which sodium nitrite was gradually added. [30b] Nitrosation of anisole was performed with sodium nitrite in a mixture of dichloromethane and trifluoroacetic acid. [30c] The product had a λ_max_ at 350 nm. Phenols were tosylated with *p*-toluenesulfonyl chloride in pyridine to afford the corresponding phenyl *p*-toluenesufonate to be used as standards in HPLC analysis of the reaction mixtures. [30d]

### Kinetic study of the basic transnitrosation of phenols in the presence of a radical trap (TEMPO)

Volumes of 50 *µ*L of 0.3 M solution of 3-metho × yphenol, 90 *µ*L of TEMPO solution, 70 *µ*L of 1,4-dio × ane, and 2760 *µ*L of pH 10.75 phosphate buffer solution were pipetted to a quartz cell. After temperature stabilization, 30 *µ*L of MeNMBS solution was added and the formation of 4-nitrosophenol monitored. A control experiment was set at the same time and the radical trap solution replaced by the same volume of buffer, which amounted to 2850 *µ*L. The same tests were made with thymol.

## Results and Discussion

**Figure 1:**
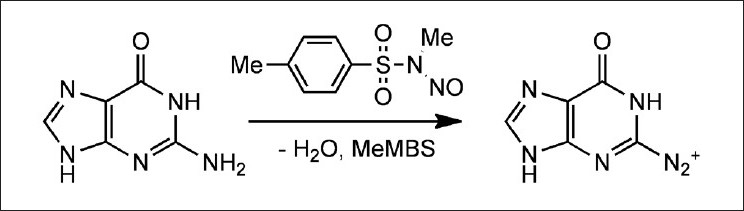
Transnitrosation step in the nitrosative deamination of guanine

The transnitrosation of DNA bases [Figure 1] was studied in acidic medium due to their low solubility at higher pH values, guanine being the least soluble of them, which limited the range of concentrations that could be used. At low pH, *N*-Nitrosobezenesulfonamides undergo hydrolysis, so a competition between both processes is expected. The observed rate constant for the consumption of MeNMBS should be the sum of the two contributions: transnitrosation (k_T_ ) and hydrolysis (k_H_ ^+^ ), according to eq. 1.

(1) $$k_{obs}\;=\;k_{T}\;\left[DNA\;base\right]\;+\;k_{H+}\;\left[H^{+}\right]\;$$

Although UV / Vis spectroscopy is broadly used to monitor the progress in organic reactions, mainly due to spectral similarities of the involved species, HPLC with diode array detection proved to be a more reliable quantitative methodology.

Reaction of MeNMBS (66.7 *µ*M) with excess guanine (174 to 348 *µ*M), corresponding to *pseudo* first-order conditions, was carried out in dioxane/water (~1 : 150), at 35°C and a pH 3 phosphate buffer. The decay of the former was monitored by HPLC analysis of the reaction mixture. The corresponding peak areas in the chromatograms were adjusted to a first-order integrated equation for each guanine concentration and the corresponding value of k_obs_ calculated.

The plot of k_obs_ against the guanine concentration proved the reaction to be independent of DNA base concentration, which meant that MeNMBS was being hydrolyzed and no transnitrosation occurred, at least to a measurable extent. The estimated value of k_H_^+^ compares to the value determined in our laboratory following the hydrolysis of MeNMBS at the same temperature by UV/Vis spectroscopy, 5.5×10 ^-2^ M ^-1^ s ^-1^ . At pH 4, although hydrolysis was slower, it still prevailed over transnitrosation.

The same procedure was applied to cytosine and adenine, but because of their higher solubilities, wider ranges of concentrations could be used: 174 *µ*M to 9.93 mM for the former and 199 *µ*M to 9.93 mM for the latter. In both cases results were the same as those obtained with guanine.

In the reaction of phenols with nitrosating agents, the major product is the corresponding 4-nitrosophenol 2, the most stable isomer, due to a tautomeric equilibrium with the quinone monoxime form. A small percentage of the 2-nitroso compound may also form, except when position 4 holds a substituent, in which case the latter is formed exclusively. When the phenol bears a methoxy substituent in position 3, and because of electronic factors, the opposite distribution of products is observed.[31]

It has been discussed whether the reaction occurs directly at the carbon atom or if the previous formation of an *O*-nitroso intermediate is involved. Casado *et al*,[19,22,23] have shown unambiguously that the reaction with nitrous acid occurs by the first of these mechanisms. However, other nitrosating agents, like nitrososulfonamides[21] and alkyl nitrites,[24] in basic media, present a different behavior. In both cases, the possibility of the formation of an unstable aryl nitrite, which, upon homolysis followed by recombination, afforded the C-nitroso product, was invoked. A similar combination between nitroso and pheno × y radicals was observed by EPR.[32]

**Figure 2:**
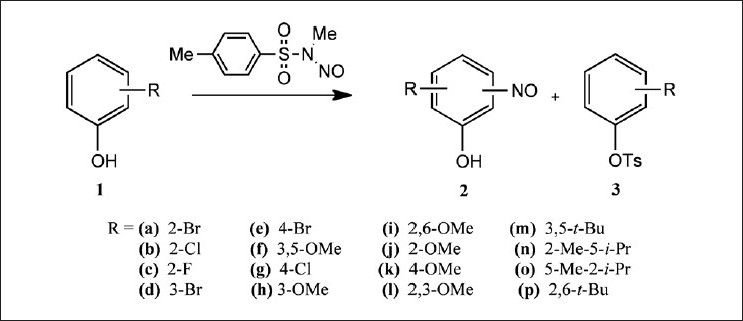
Nitroso group transfer from MeNMBS to substituted phenols

In order to further enlighten the reaction mechanism, kinetic experiments involving the transnitrosation of a series of substituted phenols 1 by *N*-Methyl-*N*-Nitroso-4-metilbenzenesulfonamide (MeNMBS) were run at 25°C in 5% dioxane / water in the pH range 9.5 – 11.5. The ambident character of the electrophile leads, in some cases, to the occurrence of the phenol *p*-toluenesulfonate 3 along with 2[Figure 2]. In such cases, the product distribution was established by the conjugation of HPLC analysis of the reaction mixture with quantification of the initial MeNMBS, using Shinn’s method.[29] The obtained results are summarized in Table 1.

Nitrosation of chloro- and bromophenols was reported to be negligible.[19] In fact, the halogenated phenols studied (1a-e and 1g) reacted very slowly with MeNMBS and the absorbance-time data deviated significantly from the best-fitting first order integrated equation. Kinetic constants estimated from the available data were in the order of 10 ^-4^ M ^-1^ .s ^-1^, which was the magnitude of the basic hydrolysis constant of MeNMBS determined in our laboratory, so a competition between the two reactions should be expected.

**Figure 3:**
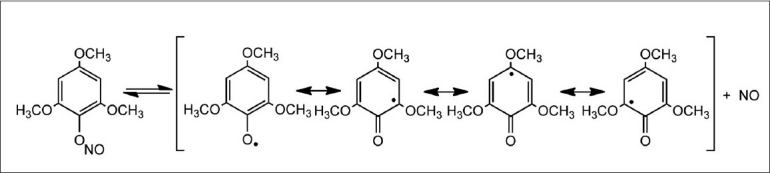
Possible stabilization of the pheno × yl radical by the metho × y groups

In the case of phenols with methoxy substituents in positions 2, 4, and / or 6 (1i-l), the corresponding nitrosophenol (2i-l) was not detected. A probable cause would be a stabilization effect of the intermediate radical, formed by homolysis of the initial aryl nitrite, preventing the recombination step [Figure 3]. Another possible explanation was the fact that methoxy substituents in these positions put a negative charge by resonance in the carbon bearing the hydroxyl group, thus destabilizing the phenoxide ion, although that effectis not very patent in the order of pK_a_ values.

As for 3,5- and 2,6-*t*-buthylphenols (1m and 1p), the former did not react due to steric hindrance and the latter was not soluble in the medium basicity media. A pH of 12.5 was needed to achieve complete solubilization, at which point hydrolysis of MNTS was much faster than transnitrosation. The fact that 2,6-*t*-buthylphenol was nitrosated by nitrous acid, but the 3,5-isomer was not, clearly emphasizes the difference in mechanism operating in acidic nitrosation and basic transnitrosation.

3,5-dimetho × y- and 3-metho × yphenols as well as carvacrol and thymol (1f, 1h, 1n, and 1o) exhibited fittable absorbance-time data and measurable rate constants. In the last two compounds, nitrosophenol (2n and 2o) was the only product, while in the first two formations, *p*-toluenesulfonate (3f and 3h) was also observed.

**Figure 4:**
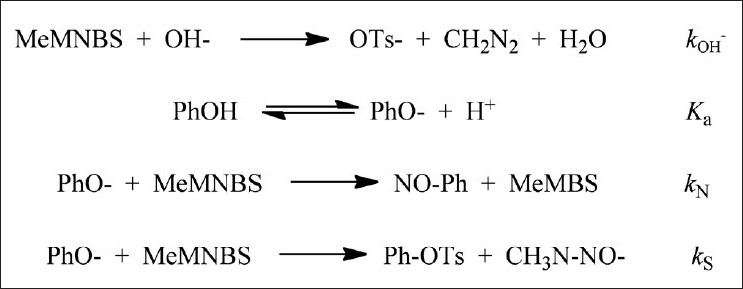
None

In a basic medium [Figure 4], MeNMBS may get hydrolyzed (k_OH_ ^-^ ), transfer its nitroso group to the phenolate ion (k_N_ ) or undergo attack by the former in its sulfonyl group (k_S_ ):

The observed rate constant for the formation of nitrosophenols should account for all the processes (eq 2):

(2) $$\begin{array}{l} k_{obs}\;=\frac{k_{Nu}K_{a}}{\left[H^{+}\right]+K_{a}}\left[PhOH\right]+k_{OH^{-}}\left[OH^{-}\right]\\ \mathrm{with}\;k_{\mathrm{Nu}}\;=\;k_{N}\;+\;k_{S}. \end{array}$$

**Figure 5:**
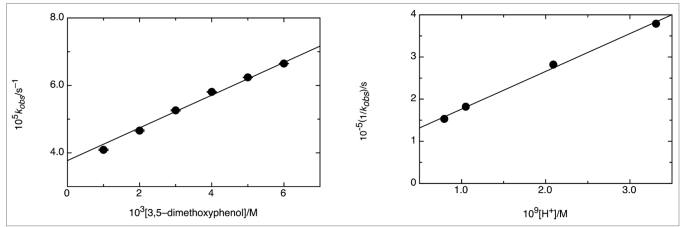
Reaction of 3,5-dimetho × yphenol with MeNMBS in 5% dio × ane / water [MNTS] = 1 × 10-4 M, 25°C. (a) First-order plot relative to phenol concentration, pH = 11.38; (b) Influence of acidity on the *pseudo*-first-order rate constant, [PhOH] = 5 × 10^-3^ M

The plot of the *pseudo*-first-order rate constant against the concentration of phenol showed that the reaction was first-order in the former. The existence of an intercept was indicative of the occurrence of hydrolysis [Figure 5 (a)]. The influence of the medium pH on the reaction rate was accounted for in the first term of the previous equation. A linear plot of such term, according to eq. 3, showed a decrease in the rate constant with increasing acidity, meaning that the phenolate ion was the active nucleophile. From the slope and intercept, values of pK _a_ consistent with those reported in the literature were found [Figure 5 (b)] and Table 1.

(3) $$\frac{1}{k_{obs}}\;=\frac{1}{K_{Nu}\left[PhOH\right]}+\frac{1}{K_{Nu}K_{a}\left[PhOH\right]}\left[H^{+}\right]$$

**Figure 6:**
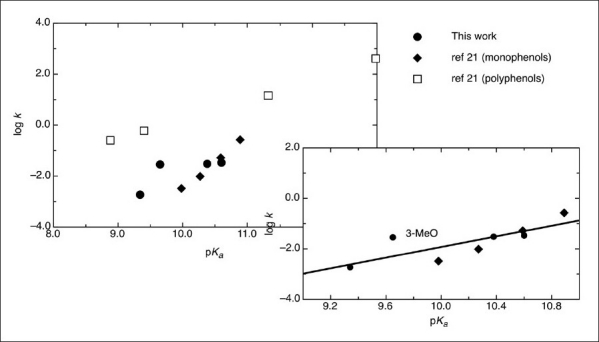
Brønsted plot for the reactions of phenolates with MeMNTS nitroso group (Insert: Brønsted relationship for monophenols)

**Figure 7:**
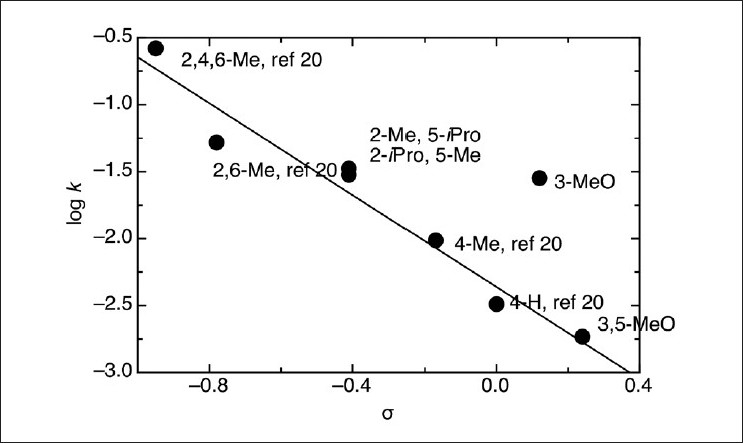
Hammett plot for the reactions of phenolates with the MeMNTS nitroso group

The existence of a linear relation between k_N_ and the pK_a_ of those phenols and four others, whose reaction with MeNMBS was reported[21] is patent in Figure 6. The 3-MeO derivative was out of the correlation, as observed by Leis *et al*.,[24] in the case of nitrosation by alkyl nitrites. The obtained value for β_nucl._ (1.4 ± 0.2), and the *P* value (-1.7 ± 0.2) obtained from the Hammett plot [Figure 7] showed that all the reactions of monophenols occurred by the same mechanism, involving the development of a positive charge at the transition state. The fact that no reaction occurred between MeNMBS and anisole (metho × ybenzene) under the same conditions, strongly suggested the involvement of the oxygen atom, with the formation of an aryl nitrite, followed by a Fisher-Hepp-like rearrangement. However, when the reaction was run in the presence of TEMPO, a radical trap, no difference in the values of k_obs_ was noticed, indicating that either radical species were not involved in the *O*-nitroso to C-nitrosophenol rearrangement or that this occurred after the rate limiting step. The choice of TEMPO as a radical trap could be tricky, as the parent compound TEMPOL could show catalytic activity in the nitrosation of phenols, namely, in reactions with peroxynitritrite.[34]

Nevertheless, absence of the kinetic influence of the radical trap supported our previous remark. In fact the possibility of reaction via the nucleophilic carbon cannot be ruled out, and this subject should be considered in further studies.
